# Association of intrauterine presence of *Lactobacillus* spp. with inflammation and pathogenic bacteria in the uterus in postpartum dairy cows

**DOI:** 10.1262/jrd.2021-023

**Published:** 2021-10-02

**Authors:** Xinyue WU, Go KITAHARA, Tetsuya SUENAGA, Kanami NARAMOTO, Satoshi SEKIGUCHI, Yoshitaka GOTO, Takeshi OSAWA

**Affiliations:** 1) Graduate School of Medicine and Veterinary Medicine, University of Miyazaki, Miyazaki 889-1692, Japan; 2) Laboratory of Theriogenology, Department of Veterinary Sciences, Faculty of Agriculture, University of Miyazaki, Miyazaki 889-2192, Japan; 3) Laboratory of Animal Infectious Disease and Prevention, Department of Veterinary Sciences, Faculty of Agriculture, University of Miyazaki, Miyazaki 889-2192, Japan; 4) Laboratory of Veterinary Microbiology, Department of Veterinary Sciences, Faculty of Agriculture, University of Miyazaki, Miyazaki 889-2192, Japan

**Keywords:** Bovine uterus, Endometritis, *Lactobacillus* spp., Pathogenic bacteria

## Abstract

The aim of this study was to clarify the influence of *Lactobacillus* spp*.* on the degree of endometrial inflammation in the postpartum period and the relationship between *Lactobacillus* spp*.* and pathogenic bacteria in the endometrium of postpartum dairy cows. Endometrial samples were collected from 41 Holstein-Friesian cows at 4 and 8 weeks postpartum using cytobrushes for polymorphonuclear neutrophil (PMN) count and bacterial culture to isolate *Lactobacillus* spp*.*, *Escherichiacoli,* and *Trueperella pyogenes*. The 4-week samples were divided into four groups (E+L+), (E+L−), (E−L+), (E−L−) according to whether endometritis was diagnosed (E+) and *Lactobacillus* spp. was isolated (L+)*.* The diagnostic criterion for cytological endometritis was > 18% PMN. The average PMN% in the E+L+ group was lower than that in the E+L-group (P < 0.05) at 8 weeks postpartum. There were no significant correlations between the number of colonies of *Lactobacillus* spp. and *E. coli* or between that of *Lactobacillus* spp. and *T. pyogenes*. *Lactobacillus* spp. could reduce PMN% in dairy cows with endometritis during the puerperal period. In conclusion, the intrauterine presence of *Lactobacillus* spp*.* may have a positive effect on uterine involution in postpartum dairy cows.

Bovine endometritis is an inflammation of the uterine epithelial cells, and bacterial infections of the endometrium can cause uterine disease in dairy cattle after parturition, leading to decreased productivity, including subfertility [1, 2]. Endometritis curbs the secretion of LH surge and inhibits postpartum follicle growth and function, which disturbs ovulation, resulting in non-pregnancy, an increase in days open, and a reduced pregnancy rate [3,4,5]. The related decrease in annual milk production due to extended days open and low reproductive performance causes substantial economic losses. Uterine diseases are estimated to cost €1.411 billion and $650 million annually in the EU and USA, respectively [2]. Therefore, these costs should be reduced or eliminated by preventing and/or treating postpartum endometritis.

As one of the main causes of endometritis, pathogenic bacteria cannot be ignored, and *Escherichia coli* and *Trueperella pyogenes* are two species that have been shown to cause this disease [6]. During 5 to 60 days postpartum, *E. coli* and *T. pyogenes* were isolated from the uterus of cows with endometritis in 49.2% and 22.5% of the animals, respectively [3]. The presence of *E. coli* early in the postpartum period results in endometrial infection by feces through the unclosed cervix [4]. *T. pyogenes* often cause high-grade uterine contamination with *E. coli* as *E. coli* products inhibit the function of neutrophils and may support the co-infection of uteri by *T. pyogenes* at later times. [6]. Additionally, *T. pyogenes* possesses a mechanism that adheres to epithelial cells. *T. pyogenes* also expresses pyolysin, which is cytolytic for immune cells, including macrophages [7].

Some species of *Lactobacillus* are resident bacteria in the vagina of healthy heifers [8]. Previous research has verified that *Lactobacillus* spp*.* release lactic acid, reduce environmental pH levels, control bacterial growth [9, 10], and produce antimicrobial compounds such as hydrogen peroxide [11, 12], and/or inhibiting the adhesion of other bacteria. Lactic acid bacteria co-cultured with cow uterine epithelial cells *in vitro* showed a positive effect on the prevention of *E. coli* infection [13]. A recent study reported that *Lactobacillus* spp. were present in both the vagina and the uterus of cows during the postpartum period [14].

Stimulation of the immune response of bovine endometrial epithelial cells by certain species of *Lactobacillus* has been shown in *in vitro* experiments; a co-culture with *L. ruminis* revealed immunomodulatory properties of the uterus [14]. Polymorphonuclear neutrophils (PMNs) leave the blood and migrate toward areas of inflammation [15, 16]. Previous reports have confirmed that bacterial infection in the uterus and endometritis induce endometrial PMN infiltration [17]. Therefore, the percentage of PMNs to all nucleated cells (PMN%) in the uterus is an important index for monitoring uterine involution and diagnosing endometritis in cows [18, 19]. However, no definitive reports are demonstrating the relationship between the spontaneous presence of *Lactobacillus* spp. and PMN% in the bovine uterus or confirming whether their presence has a positive effect on uterine involution.

The aim of the present study was to clarify the association between *Lactobacillus* spp*.* and endometrial PMN% and to elucidate the relationship between *Lactobacillus* spp*.* and pathogenic bacteria (*E. coli* and *T. pyogenes*) in the uterus of postpartum dairy cows.

## Materials and Methods

### Animals

**Fig. 1.**
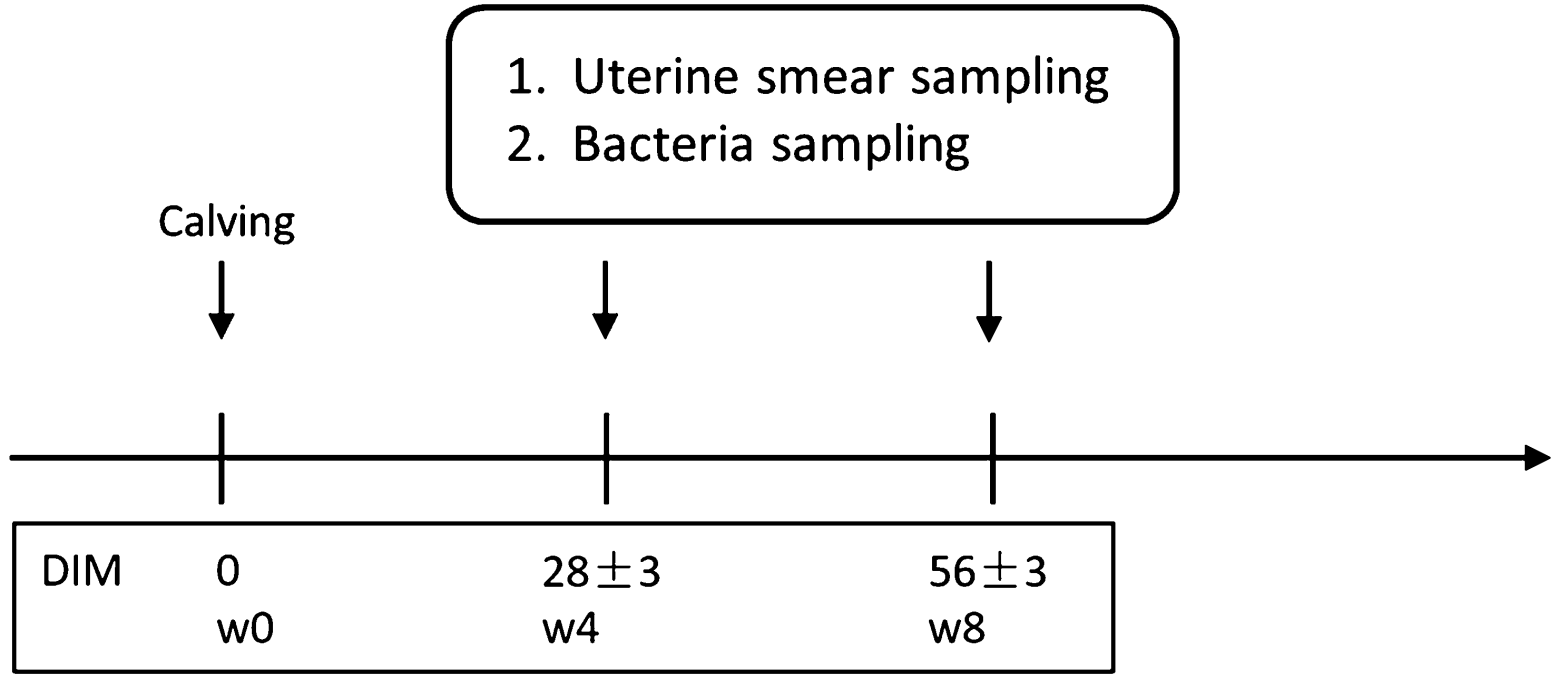
Experimental design. Holstein-Friesian cows (n = 41) undergo two procedures: 1. Uterine smear sampling: 2. Bacterial sampling in the research. DIM: days in milk.

This study involved 41 clinically healthy Holstein-Friesian cows reared on a dairy farm. The average age of the cows was 4.5 ± 1.4 years, and the average parity was 2.4 ± 1.2. The experimental period was from July 2017 to October 2018. Cytological and bacteriological examinations of the endometrium were performed at weeks 4 (28 ± 3 days; w4) and 8 (56 ± 3 days; w8) postpartum (pp) (Fig. 1

). The animals were left untreated during the experimental period.

The care and use of animals complied with local animal welfare laws, guidelines, and policies.

### Cytobrush technique

The genital area was sprayed with alcohol, the labia were gently opened, and a cytobrush (Metribrush, Fujihira Industry Co., Ltd., Tokyo, Japan), which was composed of a stainless-steel sleeve and long handle brush, was inserted into the vagina. To prevent contamination, the brush was placed in the sleeve during insertion. Then, the brush head was pushed through the cervix to the middle of the uterine body and rolled to collect the cells. Two endometrial samples were collected, and one was smeared onto two clean glass slides and fixed using 99% ethanol. The other cytobrush samples were shaken 100 times in 1 ml of physiological saline for bacterial culture. The samples were placed at 0–5°C in an icebox and transported to the laboratory within 6 h.

### Endometrial cytology

The samples fixed on the glass slide were stained using Diff-Quik (Sysmex, Kobe, Japan) staining solution, air-dried, and placed under a microscope at 400 × magnification. Two hundred nucleated cells were classified per piece. The average proportion of PMN counts over the nucleated cells on the two slides was calculated as PMN percentage (PMN%). The diagnostic criteria for cytological endometritis were > 18 PMN% [18] at w4 pp and > 4% at w8 pp [20].

### Bacteriological examination

The samples for bacteriology were serially diluted with saline (×10, ×100, ×1000, or ×100, ×10,000, and ×100,000 times dilutions), and 100 μl of each concentration of liquid was inoculated by pipette into 5% horse blood (horse whole blood defibrinated sterile, Nippon Bio-Supp. Center, Tokyo, Japan) containing SCD medium (Trypto-Soya Agar; Nissui Co., Tokyo, Japan) in duplicate for aerobic and anaerobic cultures. The media were placed in an incubator at 37°C for 48 h. To isolate *Lactobacillus* spp*.*, a non-diluted part of the bacterial sample was cultured individually on MRS agar at pH 4.5 with acetic acid (Sigma-Aldrich, St. Louis, MO, USA), and placed in an anaerobic environment in an incubator at 37°C for 48 h. Bacterial species were determined according to the microbial identification system (MALDI Biotyper; Bruker Daltonik GmbH, Bremen, Germany), and the number of bacterial colonies was calculated for each sample. Positive bacteria were defined as one or more colonies of each bacterial species detected, whereas negative bacteria were defined as no colony detected.

### Division of the experimental group

Endometrial samples were collected from 41 cows at w4 and w8 and used in the present study. According to the criterion for PMN% or isolation of *Lactobacillus* spp. at w4, the cows were classified into endometritis (E+) and non-endometritis (E–) groups, and *Lactobacillus* spp.-positive (L+) and *Lactobacillus* spp.-negative groups (L–). Therefore, the animals were divided into four categories: (E+L+), (E+L−), (E−L+), and (E−L−). To clarify how the timing of the presence of *Lactobacillus* spp*.* affected uterine involution after calving, the animals were further divided into four groups: (w4–w8–), (w4–w8+), (w4+w8–), and (w4+w8+) groups according to the presence (+) or absence (–) of *Lactobacillus* spp. at weeks 4 (w4) and 8 (w8) pp.

### Statistical analysis

The proportion of cows with endometritis between w4 and w8 within each L+ or L– group and between the L+ and L– groups was compared using Fisher’s exact test. PMN% at w4 and w8 were analyzed between the E+L+, E+L–, E–L+, and E–L– groups, and within each group using the Mann-Whitney U test. Similarly, PMN% at w4 or w8 was analyzed among w4–w8–, w4–w8+, w4+w8–, and w4+w8+ groups, and within these groups by Mann-Whitney U test. The detection rates of *E. coli* and *T. pyogene*s were compared between the *Lactobacillus* spp. isolated group and no *Lactobacillus* spp. isolated group by Chi-squared test, irrespective of the week. Spearman’s rank correlation coefficient was used to analyze the number of colonies of *Lactobacillus* spp., *E. coli*, and *T. pyogenes.* The number of *Lactobacillus* spp. colonies was compared using the Mann-Whitney U-test between cows with and without endometritis. The correlation between PMN% and the number of colonies in *Lactobacillus* spp. was also analyzed using Spearman’s rank correlation coefficient. To compare the four groups according to the presence of *Lactobacillus* spp. at weeks 4 (w4) and 8 (w8), multiple comparison analysis was conducted using pairwise Wilcoxon rank-sum tests. All statistical analyses were conducted using R software (version 4.0.5; R Core Team, Vienna, Austria). Differences were considered statistically significant at P ˂ 0.05. Values are expressed as mean ± SD.

## Results

### Association between Lactobacillus spp. and endometritis

Endometritis positivity rates in the L+ group at w4 and w8 were 30.0% (6/20) and 10.0% (2/20), respectively, and those in the L– group were 23.8% (5/21) and 23.8% (5/21), respectively (Table 1

**Table 1.** Distribution of cows with E+: endometritis ^1)^ or E–: no-endometritis at w4 or w8 and those with or without *Lactobacillus* spp. at w4 ^2)^

Weeks	*Lactobacillus* spp. at w4	E+ (n)	E– (n)	Total (n)	Positive rate (%)
w4	L+	6	14	20	30.0
	L–	5	16	21	23.8

w8	L+	2	18	20	10.0
	L–	5	16	21	23.8

^1)^ Cows with endometrial PMN% greater than 18 are diagnosed as endometritis-positive at w4, and those with endometrial PMN% greater than 4 are diagnosed as endometritis-positive at w8. ^2)^ Cows with *Lactobacillus* spp. detected at w4 are diagnosed as L+.

). There were no significant changes in the positive rate of endometritis from w4 to w8 in the L+ and L– groups.

**Fig. 2.**
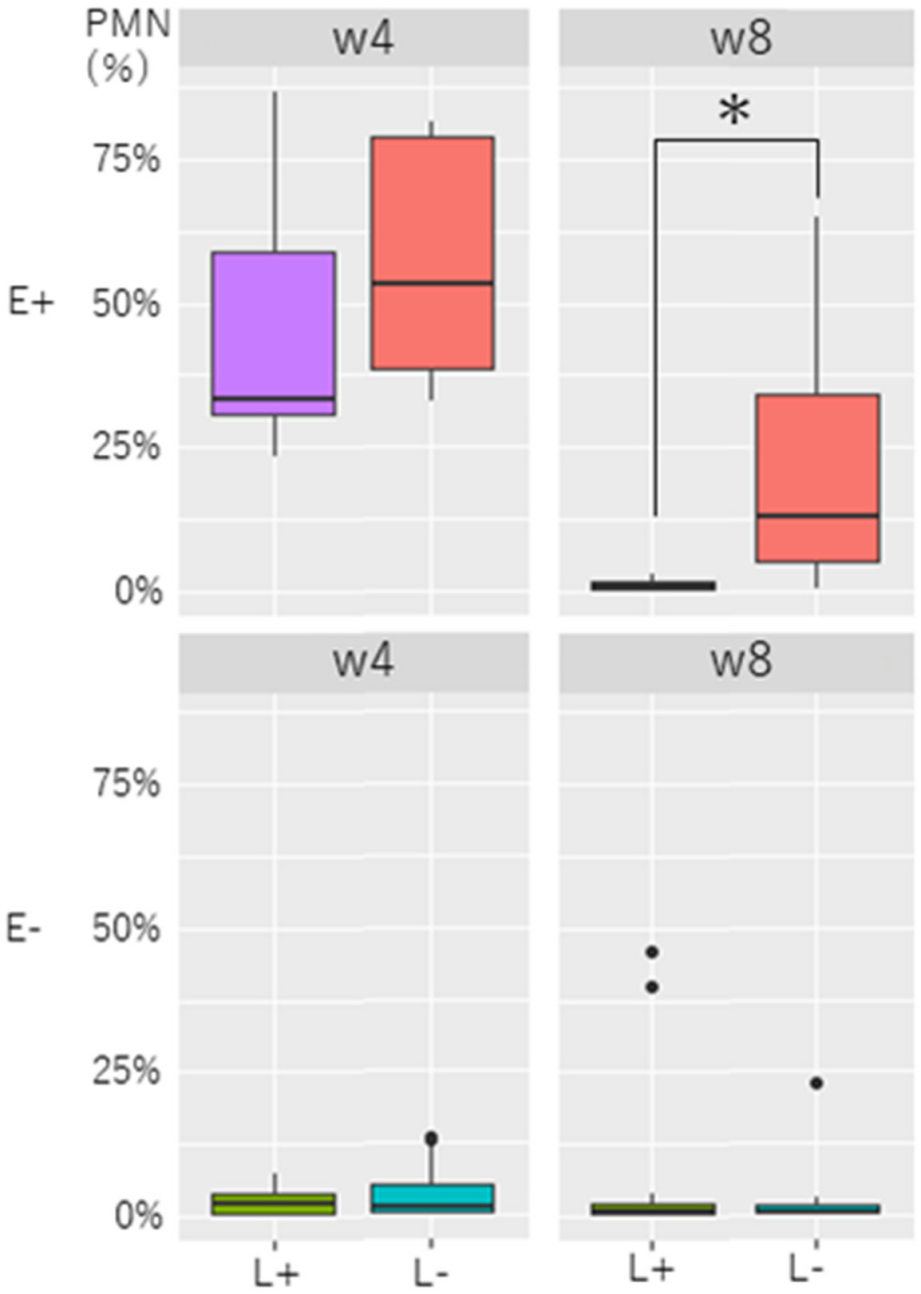
Association of *Lactobacillus* spp. and endometritis in postpartum dairy cows. PMN% in the endometrium of dairy cows with or without endometritis and with or without *Lactobacillus* spp., which are diagnosed at w4 pp, are compared at w4 and w8 pp. E+ (upper panels): These graphs show that endometritis is diagnosed at week 4 postpartum. E– (lower panels): These graphs show that endometritis is not diagnosed at week 4 postpartum. L+: Cows with *Lactobacillus* spp. L–: Cows without *Lactobacillus* spp. w4: PMN% at week 4 postpartum. w8: PMN% at week 8 postpartum. * P < 0.05.

While the average PMN% at w4 in the E+L– group (57.0 ± 22.5%) was similar (P = 0.36) to that in the E+L+ group (45.7 ± 25.2%), the average PMN% in the E+L– group (23.6 ± 26.4%) was significantly higher (P < 0.05) than that in the E+L+ group (1.1 ± 1.2%) at w8 (Fig. 2

). PMN% in the E–L– group (3.8 ± 4.9%) was not different (P = 1.0) from that in the E–L+ group (2.5 ± 2.2%) at w4, and no difference (P = 0.9) was found in PMN% between the E–L– group (2.4 ± 5.6%) and E–L+ group (7.0 ± 15.3%) at w8.

### Association of the presence of Lactobacillus spp. in the uterus with endometrial PMN%

**Fig. 3.**
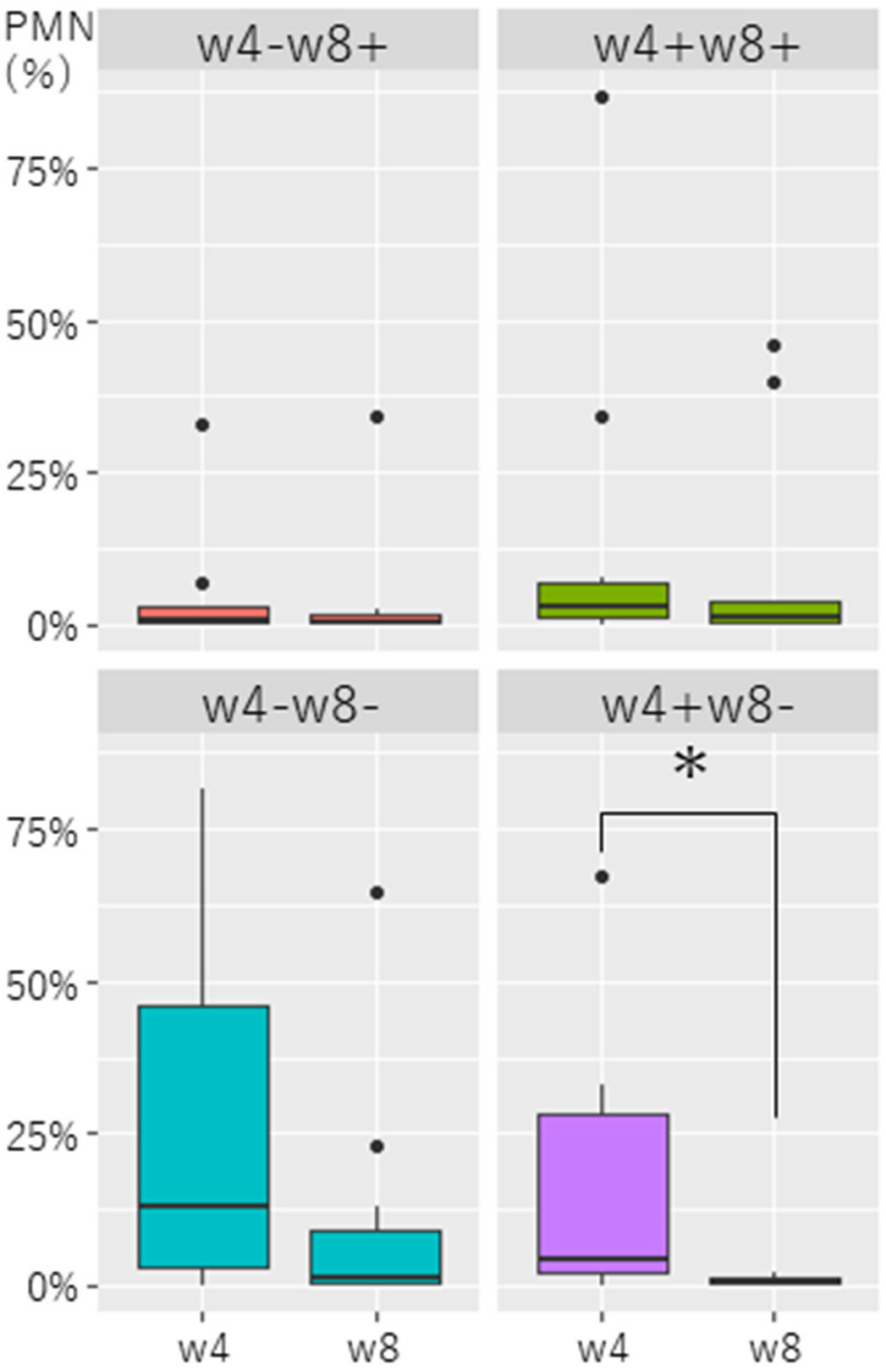
Comparison of PMN% in the endometrium of dairy cows with *Lactobacillus* spp. present cows or absent cows at week 4 or week 8 postpartum. w4–w8+: *Lactobacillus* spp. are not isolated at week 4 but are isolated at week 8 postpartum. w4+w8+: *Lactobacillus* spp. are isolated at both week 4 and week 8 postpartum. w4–w8–: *Lactobacillus* spp. are not isolated at weeks 4 and 8 postpartum. w4+w8–: *Lactobacillus* spp. are isolated at week 4 but not isolated at week 8 postpartum. * P < 0.01.

Based on the bacteriological examination results, eight cows were classified into the w4–w8+ group, 10 cows in the w4+w8+ group, 13 cows in the w4–w8– group, and 10 cows in the w4+w8– group (Fig. 3

). There was no significant difference in PMN% among the four groups at w4 (w4–w8+: 5.4 ± 11.3%; w4+w8+: 14.2 ± 27.3%; w4–w8–: 23.3 ± 29.8%; w4+w8–: 16.8 ± 21.9%) and w8 (w4–w8+: 4.9 ± 11.9%; w4+w8+: 9.6 ± 17.6%; w4–w8–: 8.9 ± 18.0%; w4+w8–: 0.9 ± 0.8%), respectively (Supplementary Fig. 1). However, PMN% in the w4+w8– group at w8 (0.9 ± 0.8%) was lower (P < 0.05) than that at w4 (16.8 ± 21.9%, Fig. 3). Moreover, while the endometritis rate in the w4–w8– group remained the same (30.8%, 4/13) from w4 to w8, that in the w4+w8– group decreased from 40% (4/10) at w4 to 0% (0/10) at w8.

### Association of the intrauterine presence of Lactobacillus spp. with pathogenic bacteria

Since both *E. coli* and *T. pyogenes* were isolated simultaneously from two of the 82 samples, the two samples were included in both *E. coli* positive and *T. pyogenes* positive categories, creating a total of 84 samples. *Lactobacillus* spp. were isolated in 39 of the 84 (46.4%) samples and were absent in the remaining 45 (53.6%). *E. coli* was present in 10 (25.6%) of the 39 samples in the *Lactobacillus* spp. isolated group, and in five (11.1%) of the 45 samples in the *Lactobacillus* spp.-absent group, and there was no significant difference in the percentage of *E. coli* positives between the two groups. Similarly, no significant difference was found in the *T. pyogenes* positive rate in the *Lactobacillus* spp. present group (six samples; 15.4%) and *Lactobacillus* spp. absent group (nine samples; 20.0%). There were no significant correlations between the number of colonies of *Lactobacillus* spp. and *E. coli* or between *Lactobacillus* spp. and *T. pyogenes*.

### Association of Lactobacillus spp. colony number with PMN%

The colony number of *Lactobacillus* spp. at w4 in the E+ group (81.4 ± 100.2) was not significantly different from that in the E– group (90.6 ± 112.7). Similarly, the colony number at w8 in the E+ group (136.7 ± 148.4) was not different from that in the E– group (85 ± 107.4). In a total of 39 samples from which *Lactobacillus* spp. were isolated at either w4 or w8, no significant change in PMN% was observed with an increase in *Lactobacillus* spp. colony number.

## Discussion

In the present study, we investigated the association between the presence of *Lactobacillus* spp*.* and the degree of endometrial inflammation and pathogenic bacteria in the endometrium of postpartum dairy cows.

Uterine epithelial cells may regenerate approximately 25 days after parturition [21]; therefore, the timing of endometrial sampling was determined at w4 (28 ± 3 days pp) to remove the effects of physiological inflammation during the puerperium period of uterine involution. In the present study, *Lactobacillus* spp. decreased the prevalence of endometritis, which corroborates previous research showing that *Lactobacillus* spp. reduced the incidence of uterine infection in periparturient dairy cows [22]. In addition, the E+L+ group had lower PMN% at w8 compared to the E+L– group. These results indicate that the presence of *Lactobacillus* spp. in cows with endometritis during the puerperium period (w4) may mitigate endometrial inflammation by w8. Some *Lactobacillus* species may release hydrogen peroxide, and lactobacilli are present in the vaginal microflora of healthy cows, where they can prevent pathogen colonization by the production of antagonistic substances such as lactic acid, hydrogen peroxide, or bacteriocins [10, 23]. This possibly explains how *Lactobacillus* spp. can inhibit the development of endometritis.

Regarding the relationship between the presence of *Lactobacillus* spp. and endometrial PMN%*,* the w4–w8– group had a high average PMN% at both w4 and w8, and the endometritis rate in the w4–w8– group remained the same (30.8%, 4/13) from w4 to w8. These results show that uterine involution stagnated during the period from w4 to w8 in cows with a high degree of inflammation and no *Lactobacillus* spp. in the endometrium at w4, since the absence of *Lactobacillus* spp. may have affected postpartum uterine involution. In contrast, the w4+w8– group was the only group with an average PMN% (16.8 ± 21.9%), which was substantially higher than the cut-off points of 4% for endometritis at w4, which then significantly decreased to the “no-endometritis” level (0.9 ± 0.8%) by w8. Additionally, the endometritis rate in the w4+w8– group decreased from 40% (4/10) at w4 to 0% (0/10) at w8. It is assumed that even if the degree of uterine inflammation is high at w4, the presence of *Lactobacillus* spp. may sufficiently reduce the degree of endometrial inflammation by w8. A previous study showed that the co-culture of *Lactobacillus* spp. with endometrial epithelial cells *in vitro* can stimulate epithelial cells to secrete immune factors [14]. Another study observed that intravaginal administration of *Lactobacillus* spp. before and after calving accelerated postpartum uterine involution in dairy cows [24]. In the present study, the w4–w8+ and w4+w8+ groups had lower average PMN% values than the other two groups between w4 and w8. It is conjectured that if the degree of endometrial inflammation was sufficiently low by w4, there would have been no additional positive effect of *Lactobacillus* spp.

Previous studies have confirmed that *Lactobacillus* spp. inhibit bacterial growth by producing lactic acid and hydrogen peroxide [12, 25, 26, 27]. Tachedjian *et al*. (2017) suggested that lactic acid is a major antimicrobial factor produced by lactobacilli [28]. Previous research has shown that *E. coli* possesses three different types of systems to resist acid stress from pH 2 to 4.5 [29]. No apparent inhibitory effect of *Lactobacillus* spp. on *T. pyogenes* and *E. coli* infections was observed in the present study. One of the reasons for this discrepancy may be due to the small sample size for the comparison of the colony number, and further research using a larger population is required to elucidate whether *Lactobacillus* spp. inhibit the growth of *E. coli* and *T. pyogenes*.

To reveal the relationship between the number of *Lactobacillus* spp*.* colonies and uterine PMN percentage, *Lactobacillus* spp. colony counts and PMN% were observed. The data showed no significant change in PMN% with an increase in *Lactobacillus* spp. colony number. These results contradict the findings of a previous report stating that lactic acid production by *L. crispatus* and *L. gasseri* inactivated the growth of several different bacterial species, including *Chlamydia trachomatis* [30], *Neisseria gonorrhoeae* [31], and *E. coli, in vitro*. In the present study, the effect of *Lactobacillus* spp. on the uterine environment in dairy cows was monitored during the middle and last stages of the puerperal period (w4 to w8 pp). Future studies are needed, focusing on these effects at an earlier stage of the puerperal period, such as week 3 or earlier postpartum. Moreover, alternative methods to determine the bacterial quantity would aid in understanding the interactions between *Lactobacillus* spp. and other bacterial species in the bovine uterus.

In conclusion, the average PMN% at 8 weeks postpartum in the E+L+ group was significantly lower than that in the E+L–group. The timing of the presence of *Lactobacillus* spp. at a certain stage after calving may have diagnostic significance. Further studies with a larger sample size are required to clarify the actual effects on fertility in the field.

## Conflict of interests

The authors declare no conflicts of interest associated with this manuscript.

## Supplementary

Supplement Figure
