# Supplementary material for: Association of intrauterine presence of Lactobacillus spp. with inflammation and pathogenic bacteria in the uterus in postpartum dairy cows
Source: J Reprod Dev. 2021 Oct 2;67(6):340–4. doi: 10.1262/jrd.2021-023 (PMC8668369; doi:10.1262/jrd.2021-023)

**Supplementary Fig. 1.**

Comparison of PMN% in the endometrium of dairy cows with *Lactobacillus* spp. present cows or absent cows at week 4 or week 8 postpartum.

w4–w8+: *Lactobacillus* spp. are not isolated at week 4 but are isolated at week 8 postpartum.

w4+w8+: *Lactobacillus* spp. are isolated at both week 4 and week 8 postpartum.

w4–w8–: *Lactobacillus* spp. are not isolated at weeks 4 and 8 postpartum.

w4+w8–: *Lactobacillus* spp. are isolated at week 4 but not isolated at week 8 postpartum.

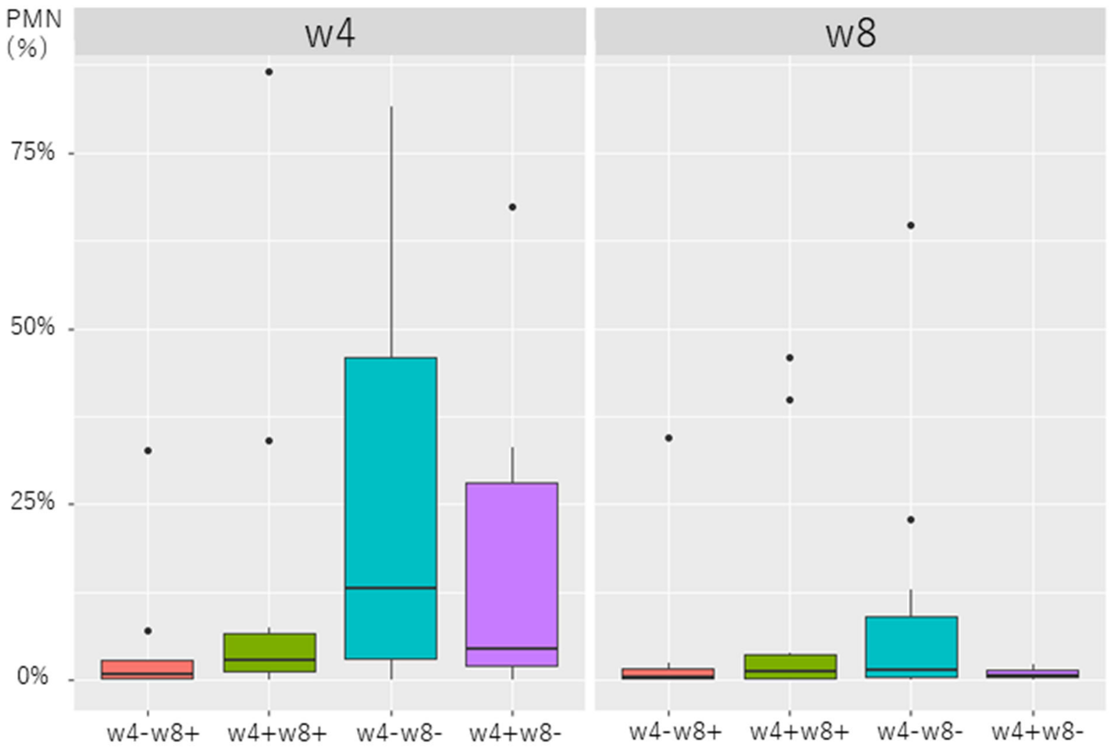

Supplement: Supplement Figure [file jrd-67-340-s001.pdf]
